# Comprehensive germline and somatic genomic profiles of Chinese patients with biliary tract cancer

**DOI:** 10.3389/fonc.2022.930611

**Published:** 2022-08-22

**Authors:** Haipeng Yu, Yan Xu, Wei Gao, Mei Li, Ji’an He, Xiaoqian Deng, Wenge Xing

**Affiliations:** ^1^ Department of Interventional Therapy, Tianjin Medical University Cancer Institute & Hospital, National Clinical Research Center for Cancer, Tianjin, China; ^2^ Tianjin's Clinical Research Center for Cancer, Tianjin, China; ^3^ Key Laboratory of Cancer Prevention and Therapy, Tianjin, China; ^4^ Department of Medical Affairs, Lifehealthcare Clinical Laboratory, Hangzhou, China

**Keywords:** biliary tract cancer, genomic profile, germline, DNA damage repair, intrahepatic cholangiocarcinoma, extrahepatic cholangiocarcinoma, gallbladder carcinoma, actionable alterations

## Abstract

**Background:**

Biliary tract cancer (BTC) is an uncommon but highly lethal malignancy with poor clinical outcomes. To promote the development of precision medicine for BTC, uncovering its genomic profile becomes particularly important. However, studies on the genomic feature of Chinese BTC patients remain insufficient.

**Methods:**

A total of 382 Chinese patients with BTC were enrolled in this study, including 71 with intrahepatic cholangiocarcinoma (ICC), 194 with extrahepatic cholangiocarcinoma (ECC), and 117 with gallbladder carcinoma (GBC). Genetic testing was performed by utilizing the next-generation sequencing (NGS) of 499 cancer-related genes and the results were compared to those of Western BTC patients (MSKCC cohorts).

**Results:**

The most prevalent genes were *TP53* (51.6%), *ARID1A* (25.9%), *KMT2C* (24.6%), *NCOR1* (17%), *SMAD4* (15.2%), *KRAS* (14.9%), *KMT2D* (14.9%), *ATM* (14.1%), and *APC* (13.9%) in Chinese BTC patients. *TP53, SMAD4*, and *APC* were more prevalent in GBC, ECC, and ICC, respectively. In addition, 10.5% of Chinese BTC patients harbored pathogenic or likely pathogenic (P/LP) germline alterations in 41 genes, which were mainly related to DNA damage repair (DDR). Additionally, the genomic features of Chinese and Western BTC tumors were similar, with the exception of the notable difference in the prevalence of *TP53*, *KRAS*, *IDH1*, *KMT2C*, and *SMAD4*. Notably, Chinese BTC patients had high prevalence (57.1%) of actionable alterations, especially for those with ECC, and half (192/382) of them had somatic DDR alterations, with the prevalence of deleterious ones being significantly higher than their Western counterparts. Twenty-three percent of patients had a higher tumor mutational burden (TMB-H, over 10 mutations/MB), and TMB was significantly higher in those with deleterious DDR alterations and/or microsatellite instability-high. The most common mutational signature in BTC patients was Signature 1, and interestingly, Signatures 1, 4, and 26 were significantly associated with higher TMB level, but not with the survival of patients who had received immunotherapy in pan-cancer.

**Conclusion:**

Our study elaborated the distinct germline and somatic genomic characteristics of Chinese BTC patients and identified clinically actionable alterations, highlighting the possibility for the development and application of precision medicine.

## Introduction

Biliary tract cancer (BTC) is an aggressive, heterogeneous biliary epithelial neoplasm that accounts for 1% of adult cancers and 3% of adult gastrointestinal malignancies worldwide (1, 2). It is mainly composed of three subtypes, including intrahepatic cholangiocarcinoma (ICC), extrahepatic cholangiocarcinoma (ECC), and gallbladder carcinoma (GBC) (3). Although BTC is rare in Western countries, its incidence has been increasing in recent years, mainly due to the ICC (4, 5). Patients with BTC have a poor prognosis, with a 5-year survival of approximately 5%–15% only (6, 7). Radical surgical resection is the primary curative treatment; however, as BTC is commonly asymptomatic in its early stages and lacks specific and robust biomarkers, approximately 60%–70% of BTC patients are diagnosed with locally advanced unresectable disease (8–10). In addition, during exploratory laparotomy, a significant proportion of patients initially diagnosed with localized and resectable disease at diagnosis were subsequently confirmed to be unresectable (11). Moreover, even after radical surgery, the recurrence rate is still above 70% (12). Hence, there is an urgent need to comprehensively understand the genomic feature and thus develop novel biomarkers for early detection and providing matched therapeutic approaches for BTC.

For advanced BTC patients, there are other treatment options including chemotherapy, targeted therapy, and immunotherapy, of which gemcitabine combined with cisplatin has become the standard first-line treatment (13). For patients who harbor actionable mutations, targeted therapy is superior to chemotherapy in the second line, especially pemigatinib or infigratinib for treating *FGFR2* fusion or rearrangement (14, 15) and ivosidenib for *IDH1* mutation (16), which have been approved by the U.S. Food and Drug Administration (FDA). Pan-cancer therapies, such as larotrectinib and entrectinib for *NTRK* rearrangement tumors (17, 18), and dabrafenib combined with trametinib for treating *BRAF V600E*-mutated patients (19, 20), could also have promising efficacy against BTC. On the other hand, FDA has approved pembrolizumab to treat patients with unresectable or metastatic, MSI-H or mismatch repair-deficient (dMMR) BTC; however, only an extremely low number of patients would benefit from it because of the rare prevalence of MSI-H/dMMR in BTC (below 2%) (10, 11). The overwhelming response rates and clinical benefits of immune checkpoint inhibitor (ICI) monotherapy for unselected BTC patients are generally poor (9). To tailor matched therapy for BTC patients, precision medicine strategies relying on novel technologies such as next-generation sequencing (NGS) are particularly crucial. Moreover, it is necessary to explore the genomic differences between BTC with different race or ethnicity to obtain a comprehensive understanding of BTC.

In this study, next-generation sequencing (NGS) was performed on tissue or blood samples from 382 Chinese patients with BTC to characterize their genomic features. The genomic landscape of BTC was further clarified by comparing the genetic differences between different subtypes of BTC and between Chinese and Western patients.

## Materials and methods

### Patients and samples

This study retrospectively screened 382 Chinese patients diagnosed with BTC at Tianjin Medical University Cancer Institute and Hospital from January 2018 to October 2021, including 71 ICC patients, 194 ECC patients, and 117 GBC patients. Of these, 272 patients provided tumor tissue samples and 110 patients provided plasma samples. Informed consent for tumor analysis was obtained according to protocols approved by the Tianjin Medical University Cancer Institute and Hospital ethics committee.

### Sample preparation and genetic testing

Before DNA extraction, serial sections of the tissue were performed to assess the content and percentage of the tumor. Only samples with tumor purity over 20% on histopathological assessment were eligible for DNA extracting and sequencing. The tumor tissues and peripheral blood mononuclear cells (PBMC) were collected to extract DNA using the DNeasy Blood & Tissue Kit (Qiagen) under the manufacturer’s instructions. Meanwhile, circulating cell-free DNA (cfDNA) was extracted from plasma using the QIAamp Circulating Nucleic Acid Kit (Qiagen) following the protocol of the manufacturer. The purified gDNA and cfDNA were then quantified using the Qubit 3.0 Fluorometer and StepOnePlus System (both from ThermoFisher Scientific). For the matched germline and tumor samples, 100 ng of DNA was sheared with a Covaris E210 system (Covaris) to get 200-bp fragments and then underwent library preparation using the Accel-NGS 2S DNA Library Kit (Swift Biosciences) and the xGen Lockdown Probes kit (IDT). The custom xGen Lockdown probe was synthesized by IDT, Inc. for the exons and selected intronic regions of 499 genes (**Supplementary Table 1**). The prepared library was quantified using the Qubit 3.0 Fluorometer, and quality and fragment size were measured using Agilent 2100 Bioanalyzer (reference fragment size: 280–350 bp; DNA quality: 0.5–50 ng/μl). Samples underwent paired-end sequencing on an Illumina Novaseq 6000 platform (Illumina) with a paired-end 2×150-bp read length. Median coverage of 1,831.61× (range, 352.87, 4,473.51), 4,503.82× (1,765.89, 9,500.52), and 282.86× (62.93, 1,017.54) was achieved for tumor gDNA, plasma cfDNA, and PBMC gDNA, respectively.

### Data processing

Raw sequencing data were aligned to the reference human genome (UCSC hg19) through Burrows-Wheeler Aligner and produced a binary alignment/map (BAM) file. After the duplicate removal and local realignment, the Genome Analysis Toolkit (GATK) and lofreq were utilized for single-nucleotide variation (SNV), short insertions/deletions (indels) calling. Variants were then annotated using the ANNOVAR software tool.

### Germline variants annotation

Candidate variants identified in the gDNA from buffy coat fraction aliquots were determined as the valid germline variants (**Supplementary Table 2**) for further analysis if they met the following criteria: (1) the Allele frequency (AF) was beyond 30%; (2) supporting reads of the allele and variant were at least 15 and 8, respectively; (3) the frequency of the variants was below 1% in the public germline variants datasets, including 1000 genomes, ExAC, and gnomAD; (4) the variants were not synonymous SNV; (5) the variants were in the exon and or splicing site; and (6) the variants were not present in the in-house repeat sequence database.

Then, the interpretation of germline variants followed the standards and guidelines of American College of Medical Genetics and Genomics and the Association for Molecular Pathology (ACMG/AMP) and was independently reviewed by two genetic consultants.

### Somatic variants annotation

The variant identified in the cfDNA or tumor tissue sample was defined as the valid somatic variant (**Supplementary Table 3**) if it met all the following standards: (1) AF was at least 1%; (2) AF was beyond three times than the AF of the same variant identified in the matched PBMC; (3) supporting reads of the allele and variant for ctDNA sample were at least 500 and 10, respectively; (4) supporting reads of the allele and variant for tumor sample were at least 150 and 5, respectively; (5) the number of forward or reverse strands supporting the allele alteration was at least 5; (6) strand bias at this position was below 60; (7) the frequency of the variant was below 1% in the public germline variants datasets, including 1000 genomes, ExAC, and gnomAD; (8) the variant was not synonymous (SNV); (9) the variant was in the exon and or splicing site; and (10) the variant was not present in the in-house repeat sequence database. Meanwhile, the current study did not involve the assessment of *FGFR2* rearrangement.

Then, the interpretation of the function of each somatic alteration was conducted by utilizing the OncoKB database (https://www.oncokb.org/#/) and cbioportal website (https://www.cbioportal.org/visualize).

### Tumor mutational burden and microsatellite instability calculation

Tumor mutational burden (TMB) score was calculated based on published and widely applied method (21), and samples with TMB value over 10 mutations/Mb were regarded as TMB-high (TMB-H). Meanwhile, the microsatellite instability (MSI) status of the tumor tissue was determined by using mSINGS method (22), and 199 tumor tissue samples were successfully investigated.

### Identification and classification of actionable alterations

The actionabilities of genetic alterations were determined based on the OncoKB (https://www.oncokb.org/) database and the cbioportal website (https://www.cbioportal.org/), which takes into account guidelines and recommendations from the Food and Drug Administration (FDA), the National Comprehensive Cancer Network (NCCN), and the medical literature (23). All actionable alterations were classified as levels 1, 2, 3, and 4.

### Publicly available data

The clinical and genomic data of Western BTC cohorts (Cholangiocarcinoma (MSK, Clin Cancer Res 2018) and GBC cohort [Gallbladder Cancer (MSK, Cancer 2018)] downloaded from cBioProtal were used for comparisons. Potentially actionable alterations were determined using the aforementioned method. Only the shared genes in Chinese and Western BTC cohorts were compared to uncover the genomic difference.

### Statistical analysis

Statistical Program for Social Sciences 25.0 (SPSS 25.0) and GraphPad Prism 8.0 were used for statistical analysis and graph drawing, respectively. Chi-square test and Fisher test were used to compare the significant difference of alteration in different subtypes or cohorts. Two-sided *p*-value less than 0.05 was considered statistically significant. The alteration spectrum was made by R software (https://www.r-project.org/).

## Results

### Patient characteristics

A total of 382 samples from Chinese patients with BTC were collected in this study, and **Table 1** shows the clinicopathological characteristics. There was an equal gender proportion, and 47.4% (181/382) of patients were female and 52.6% (201/388) were male. The median age of enrolled patients was 62 (range 32 to 89), and 56.5% (216/382) of the patients were in advanced stages (III–IV). For samples under genetic testing, 71.2% (272/382) were tumor tissue and 28.8% (110/382) were plasma.

**Table 1:** Clinicopathologic characteristics of the study population.

Characteristics	ICC(*n* = 71)	ECC(*n* = 194)	GBC(*n* = 117)	All(*n* = 382)
**Age**				
Median (range)	62 (33–84)	73 (32–84)	64 (34–89)	62 (32–89)
**Gender**				
Female	31 (43.7%)	82 (42.3%)	68 (58.1%)	181 (47.4%)
Male	40 (56.3%)	112 (57.7%)	49 (41.9%)	201 (52.6%)
**Stage**				
I	7 (9.9%)	8 (4.1%)	7 (6.0%)	22 (5.8%)
II	20 (28.2%)	77 (39.7%)	37 (31.6%)	134 (35.0%)
III	14 (19.7%)	32 (16.5%)	23 (19.7%)	69 (18.1%)
IV	29 (40.8%)	73 (37.6%)	45 (38.5%)	147 (38.5%)
NA	1 (1.4%)	4 (2.1%)	5 (4.2%)	10 (2.6%)
**Sample type**				
Tissue	47 (66.2%)	139 (71.6%)	86 (73.5%)	272 (71.2%)
Plasma	24 (33.8%)	55 (28.4%)	31 (26.5%)	110 (28.8%)
**TMB state**				
TMB-H	9 (12.7%)	48 (24.7%)	31 (26.5%)	88 (23.0%)
TMB-L	36 (50.7%)	89 (45.9%)	45 (38.5%)	170 (44.5%)
NA	26 (36.6%)	57 (29.4%)	41 (35.0%)	124 (32.5%)
**MSI state**				
MSI-H	2 (2.8%)	2 (1.0%)	1 (0.9%)	5 (1.3%)
MSI-L/MSS	37 (52.1%)	97 (50.0%)	52 (44.4%)	186 (48.7%)
NA	32 (45.1%)	95 (49.0%)	64 (54.7%)	191 (50.0%)

NA, Not Available; TMB-H, Tumor Mutation Burden-High; TMB-L, Tumor Mutation Burden-Low; MSI-H, Microsatellite Instability-High; MSI-L, Microsatellite Instability-Low; MSS, Microsatellite Stability; ICC, intrahepatic cholangiocarcinoma; ECC, extrahepatic cholangiocarcinoma; GBC, gallbladder carcinoma.

### Somatic genomic alterations landscape in Chinese BTC patients

**Figure 1:**
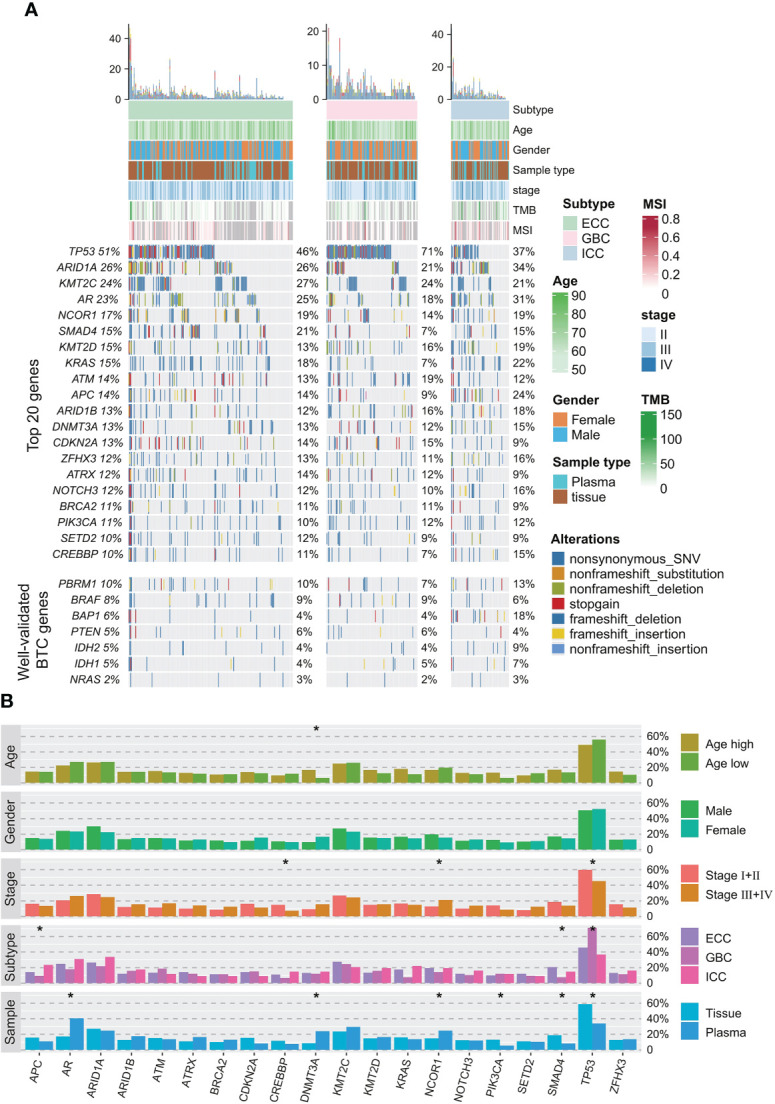
Somatic alteration profile of 382 BTC patients. **(A)** Mutation profiles of frequently mutated and well-validated mutated BTC-related genes. Relevant clinicopathological characteristics of 382 patients are shown on the top, total mutation frequencies in the cohort are shown on the left, and mutation frequencies in each histological subtype are shown on the right. **(B)** Correlations between somatic alterations and clinical phenotypes (age, gender, stage, histological subtype, and sample type) in the entire cohort. *p < 0.05. BTC, biliary tract cancer.

A total of 96.3% (368/382) of patients in our cohort had valid cancer-related somatic alterations. *TP53* (51.6% of patients), *ARID1A* (25.9%), *KMT2C* (24.6%), *NCOR1* (17%), *SMAD4* (15.2%), *KRAS* (14.9%), *KMT2D* (14.9%), *ATM* (14.1%), and *APC* (13.9%) were the most prevalent genes (**Figure 1A**). Although *NCOR1* and *KMT2C/D* alterations were relatively common in this study (**Supplementary Figure 1**), there were few studies investigating on their oncogenic role in BTC. Previous studies have found that *NCOR1* and *KMT2C/D* play oncogenic roles in bladder cancer (24), colorectal cancer (25), prostate cancer (26, 27), and lymphomagenesis (28). In addition, other important BTC genes, including *PBRM1* (10%), *BRAF* (8%), *BAP1* (6%), *PTEN* (5%), *IDH1* (5%), *IDH2* (5%), and *NRAS* (2%), were also identified with a relatively low prevalence in our cohort (**Figure 1A**). The most common type of alteration was non-synonymous variant (SNV). By correlating genomic alterations with clinical characteristics of BTC patients, we found that some genomic alterations had significant histological subtype preference (**Figure 1B**). For instance, *TP53* alterations were more prevalent in the GBC; *SMAD4* alterations were more prevalent in the ECC, but *APC* alterations were mainly enriched in the ICC instead. Meanwhile, *TP53* and *CREBBP* alterations were more enriched in BTC patients with early stages disease. In contrast, *NCOR1* alterations were more likely to occur in advanced disease. Additionally, *DNMT3A* was significantly enriched in older patients.

### Germline alterations in Chinese BTC patients

**Figure 2:**
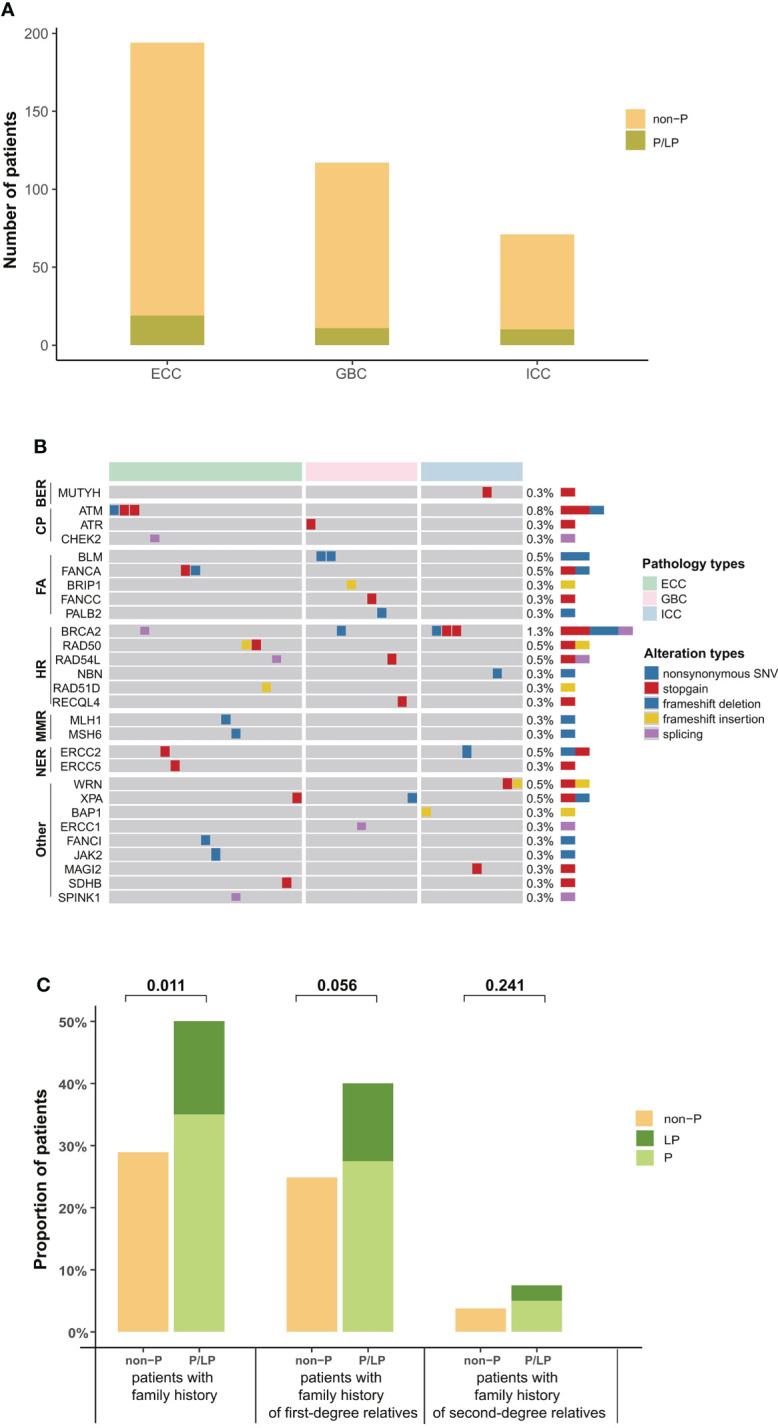
Distribution of P/LP germline alterations in 40 BTC patients. **(A)** Bar plots indicated the prevalence of P/LP germline alterations in each histological subtype (green). **(B)** P/LP germline altered genes and DDR pathways in 40 BTC patients are shown on the left, and the prevalence are shown on the right. **(C)** Family history of cancer in BTC patients with and without P/LP germline alteration. First-degree relatives include parents, children, brothers, and sisters, and other relatives are second-degree relatives. P/LP, pathogenic or likely pathogenic; BTC, biliary tract cancer; DDR, DNA damage repair.

**Figure 3:**
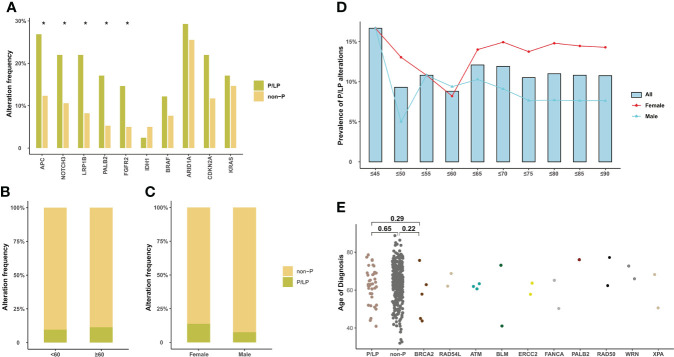
Somatic alteration characteristics of BTC patients with P/LP germline alterations and the correlation of P/LP germline alterations with clinical phenotypes. **(A)** Commonly somatic alterations in BTC patients with and without P/LP germline alteration. *p < 0.05. The correlation between P/LP germline alterations and **(B)** age or **(C)** gender. **(D)** Frequency of P/LP germline alteration in patients of different ages (372 patients with age information). The bar plot and lines show the frequency of P/LP germline alteration in patients under a certain age (bar) and frequency in female and male patients (lines). **(E)** The panel shows the age at diagnosis of patients without P/LP germline alterations and patients with different P/LP germline genes. BTC, biliary tract cancer; P/LP, pathogenic or likely pathogenic.

In addition, 10.5% (40/382) of BTC patients in our cohort were identified with pathogenic or likely pathogenic (P/LP) germline alterations, mainly in *BRCA2* (*n* = 5, 1.32%), *ATM* (*n* = 3, 0.79%), *RAD54L*, *BLM*, and *ERCC2* (*n* = 2, 0.53%) (**Figure 2A**). The prevalence of P/LP germline alterations was not significantly different among three subtypes (ECC vs. ICC vs. GBC: 5.0% vs. 2.6% vs. 2.9%) (**Figure 2B**). Only two patients (0.53%) were identified with P/LP alterations in mismatch repair pathway. In this study, 119 (31.2%) BTC patients reported a family cancer history, of which 16.81% (20/119) patients harbored P/LP germline alterations. By comparison, we found that there were significantly more BTC patients with a family history in the P/LP group than in the non-P group (50.0% vs. 28.9%, *p* = 0.011, **Figure 2C**). The most prevalent tumor types of the first- or second-degree relatives of BTC patients with P/LP germline mutations were lung (9.8%) and liver cancer (7.3%). Among BTC patients with both P/LP germline alterations and positive family cancer history, *BRCA2* (20%, 4/20) was the most prevalent. Interestingly, the frequency of *APC* (26.8% vs. 12.3%, *p* = 0.017), *NOTCH3* (22.0% vs. 10.6%, *p* = 0.041), *LRP1B* (22.0% vs. 8.2%, *p* = 0.010), *PALB2* (17.1% vs. 5.3%, *p* = 0.010), and *FGFR2* (14.6% vs. 5.0%, *p* = 0.027) somatic alterations in the P/LP group was significantly higher than those in the non-P group (**Figure 3A**). In addition, some actionable altered genes frequently studied in BTC and those with high prevalence in this study, such as *BRAF*, *ARID1A*, *CDKN2A*, and *KRAS* somatic alterations, were also relatively more prevalent in the P/LP group (**Figure 3A**). Although there was no significant correlation between P/LP germline alterations and patients’ age (**Figure 3B**) or gender (**Figure 3C**), we found a trend in our BTC cohort that germline alterations were more common in younger patients, consistent with reports in Western BTC populations (29). The prevalence of P/LP germline alterations in patients under the age of 45 was the highest (13.6%) but not significantly differed from that in patients diagnosed at older ages (**Figure 3D**). This trend may be mainly driven by patients with germline alterations in *BRCA2*, who were younger than those without germline alterations (median of 58 vs. 64, *p* = 0.22), or with other germline alterations (**Figure 3E**). However, due to the limitation in the sample size, especially those with germline variants, the difference did not reach statistical significance.

### Differences in genomic alterations between Chinese and Western BTC patients

**Figure 4:**
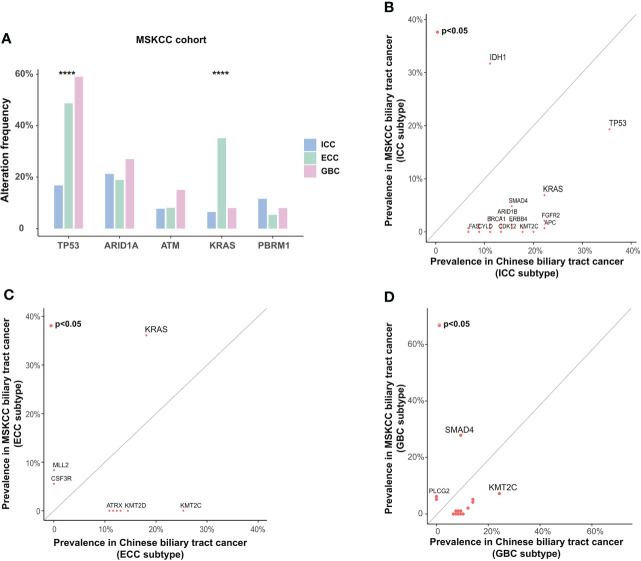
Differences in somatic alterations between Chinese and Western BTC patients. **(A)** Differences in somatic alterations between histological subtypes in the Western MSKCC cohort. ****p < 0.0001. Comparison of the prevalence of somatic alterations identified in **(B)** ICC, **(C)** ECC, and **(D)** GBC subtypes between the Chinese cohort and the Western MSKCC cohort. BTC, biliary tract cancer; MSKCC, Memorial Sloan Kettering Cancer Center; ICC, intrahepatic cholangiocarcinoma; ECC, extrahepatic cholangiocarcinoma; GBC, gallbladder carcinoma.

To determine whether there were differences in genomic characteristics between Chinese and Western BTC patients, we compared the genomic alterations in our cohort with the corresponding data in the MSKCC cohort (structural variants in *FGFR2* were excluded). We first analyzed the differences in genetic alterations among different subtypes in the MSKCC cohort (**Figure 4A**). The results showed that *TP53* alterations were more prevalent in GBC patients (*p* < 0.0001), which is inconsistent with our previous findings. In addition, the prevalence of *KRAS* also significantly differed among different subtypes, mainly enriching in the ECC (*p* < 0.0001). Subsequently, we compared the differences in genomic alterations of BTC between the Chinese cohort (tumor samples) and the MSKCC cohort. For ICC patients, the prevalence of *TP53* (35.6% vs. 16.8%, *p* = 0.012) and *KRAS* (22.2% vs. 6.5%, *p* = 0.004) in our cohort was significantly higher than that in the MSKCC cohort, whereas the frequency of *IDH1* (11.1% vs. 29.0%, *p* = 0.018) alteration was significantly lower (**Figure 4B**). The prevalence of *IDH1* was not significantly different in ECC or GBC patients between Chinese and Western cohorts, and it was highest in ICC patients in both the Chinese and the MSKCC cohorts. For ECC patients, the prevalence of *KRAS* in the MSKCC cohort was nearly twice that of our cohort (18.1% vs. 35.1%, *p* = 0.041, **Figure 4C**). In addition, *KMT2C* (23.7% vs. 7.0%, *p* = 0.002) alterations were more prevalent in GBC patients from Chinese cohort than those from the MSKCC cohort, while the prevalence of *SMAD4* (10.5% vs. 27.0%, *p* = 0.007) was significantly lower (**Figure 4D**). When analyzed based on mutational data from plasma samples, patients in our cohort also had a significant difference in genetic alterations compared with those in the MSKCC cohort, which were similar to the results described above (**Supplementary Figures 2A–C**). Except for the MSKCC cohort, we also compared the genomic feature between our cohort and the TCGA cohort or another Chinese (Shanghai, Nat Commun 2014) cohort (**Supplementary Figures 2D–F**). By comparison with the TCGA cohort, the prevalence of *IDH1* and *TP53* remained significantly different with the results in our cohort, consistent with the findings from the comparison with the MSKCC cohort. Comparing the genomic alterations across another Chinese cohort (Shanghai, Nat Commun 2014) and our cohort revealed no significant difference in those genes, demonstrating the consistency between the same ethnic groups. It should be noted, however, that these results may still be potentially biased due to variations in sample size and sequencing methods.

### Actionable genomic alterations in Chinese BTC patients

**Figure 5:**
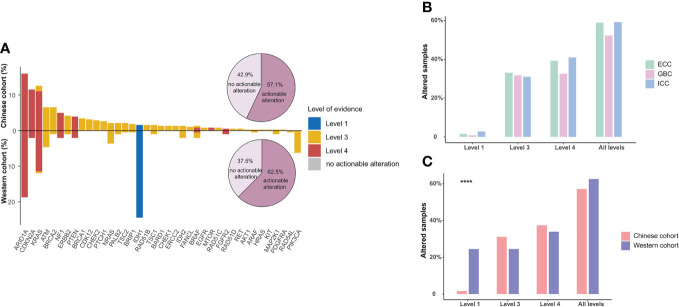
Actionable alterations in BTC patients. **(A)** Comparison of the actionable alterations between Chinese and Western BTC patients. Bar plots show the respective prevalence of actionable alterations in this study, and pie plots show the proportion of patients with actionable alterations. **(B)** The distribution of patients with actionable alterations in the Chinese cohort. **(C)** Comparison of the distribution of patients with actionable alterations in different levels in Chinese and Western cohorts. ****p < 0.0001. BTC, biliary tract cancer.

Based on the OncoKB levels of evidence, 378 actionable alterations were identified in 218 (57.1%) patients from the Chinese BTC cohort (structural variants of *FGFR2* were excluded), mainly including *ARID1A* (16.0%), *KRAS* (12.6%), and *CDKN2A* (11.5%). Next, we compared the prevalence of actionable alterations between Chinese and Western BTC patients and found that there was no significant difference in the prevalence of actionable alterations between these cohorts (62.5% vs. 57.1%, *p* > 0.05, **Figure 5A**). However, nearly a quarter of patients in the Western cohort harbored actionable alteration in *IDH1*, so the proportion of patients with level 1 actionable alteration was significantly higher than that in the Chinese cohort (24.5% vs. 1.6%, *p* < 0.0001, **Figures 5A,B**). In addition, independent of the levels of evidence, the proportion of actionable alterations was comparable among ICC, ECC, and GBC subtypes in the Chinese BTC cohort (**Figure 5C**).

### DNA damage repair, TMB, and MSI

**Figure 6:**
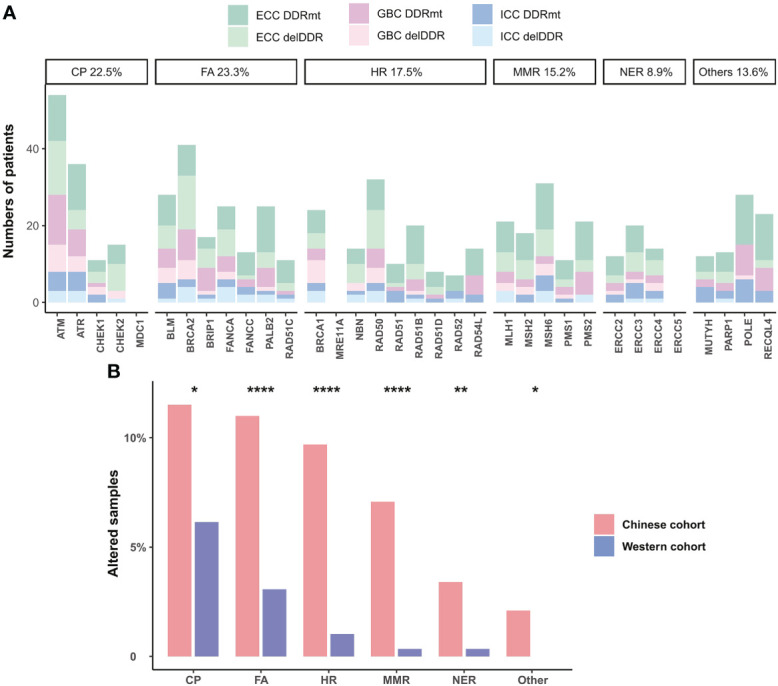
DDR alterations in BTC patients. **(A)** Distribution of somatic alterations in specific DDR pathways of ICC, ECC, and GBC subtypes. Dark colors are non-deleterious DDR alterations, and light colors are deleterious DDR (delDDR) alterations. **(B)** Comparison of the prevalence of delDDR in different DDR pathways in Chinese and Western cohorts. *p < 0.05; **p < 0.01; ****p < 0.0001. DDR, DNA damage repair; ICC, intrahepatic cholangiocarcinoma; ECC, extrahepatic cholangiocarcinoma; GBC, gallbladder carcinoma; delDDR: deleterious DDR.

Except for TMB and MSI, which are FDA-approved and widely applied biomarkers for predicting immunotherapy response in pan-cancer, DDR alteration has also been identified as a novel biomarker associated with improved efficacy. Therefore, we further assessed the DDR alterations, TMB, and MSI status in Chinese BTC patients. Somatic DDR gene alterations occurred in half (192/382) of Chinese BTC patients, including a total of 32 DDR genes that covered the six major DDR functional pathways. The most altered pathway was Fanconi anemia (FA, 23.3%), followed by checkpoint (CP, 22.5%), homologous recombination (HR, 17.5%), and mismatch repair (MMR, 15.2%) (**Figure 6A**). *ATM* was the most common DDR gene alteration in the three different subtypes of BTC, followed by *BRCA2*, *ATR*, *RAD50*, *MSH6*, and *POLE* (**Figure 6A**). We compared deleterious DDR (delDDR) alterations in Chinese and Western BTC patients and found that all six DDR pathways were more altered in the Chinese BTC cohort than in the Western MSKCC cohort (*p* < 0.05, **Figure 6B**).

**Figure 7:**
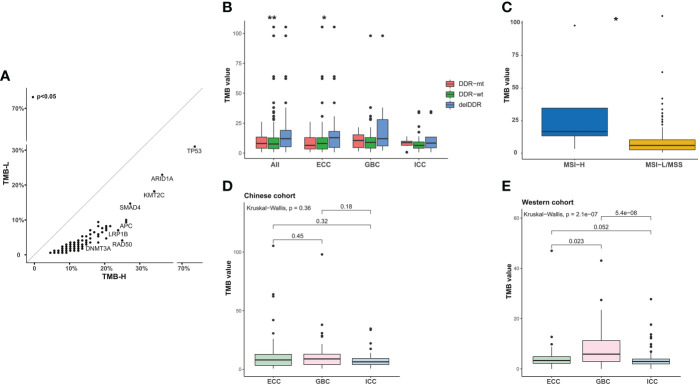
Analysis of TMB in BTC patients. **(A)** Scatter plots of the prevalence of altered genes in TMB-H and TMB-L samples from the Chinese cohort. TMB-H, TMB value ≥10 mutations/Mb; TMB-L, TMB value < 10 mutations/Mb. **(B)** Comparison of the TMB in patients with delDDR, DDR-mt, and DDR-wt. *p < 0.05; **p < 0.01. **(C)** Correlation between TMB value and MSI value. According to the median MSI value of BTC patients, patients were divided into the MSI-H group and the MSI-L/MSS group. *p < 0.05. **(D, E)** Differences in TMB values between ICC, ECC, and GBC subtypes in the Chinese and Western cohorts. TMB, tumor mutational burden; TMB-H, high TMB value; TMB-L, low TMB value; DDR, DNA damage repair; delDDR, deleterious DDR; DDR-mt, DDR mutation; DDR-wt, DDR wild type; MSI, microsatellite instability; ICC, intrahepatic cholangiocarcinoma; ECC, extrahepatic cholangiocarcinoma; GBC, gallbladder carcinoma.

Of the 382 patients with BTC, 23.0% (88/382) had a high tumor mutation burden (TMB ≥10 mutations/Mb, TMB-H). Enrichment analysis showed that *TP53*, *ARID1A*, *KMT2C*, and *SMAD4* were more prevalent in TMB-H patients (**Figure 7A**). In the meantime, TMB levels in patients with delDDR alterations were significantly higher than those in patients with non-deleterious DDR mutations (DDR-mt) and DDR wild type (DDR-wt), especially in the histological subtype of ECC (**Figure 7B**). In addition, only 2.51% (5/199) of Chinese BTC patients were microsatellite instability-high (MSI-H) in our cohort and the rest of the 186 patients were microsatellite instability-low or microsatellite instability stable (MIS-L/MSS). Despite the small number of MSI-H patients, it was found that these patients had a significantly higher TMB level (**Figure 7C**). Additionally, although median TMB values were not significant among different histological subtypes in our BTC cohort, median TMB values were relatively highest for GBC subtypes (**Figure 7D**), which was in concordance with the result in the Western MSKCC cohort (**Figure 7E**).

### Mutational signatures in BTC patients

**Figure 8:**
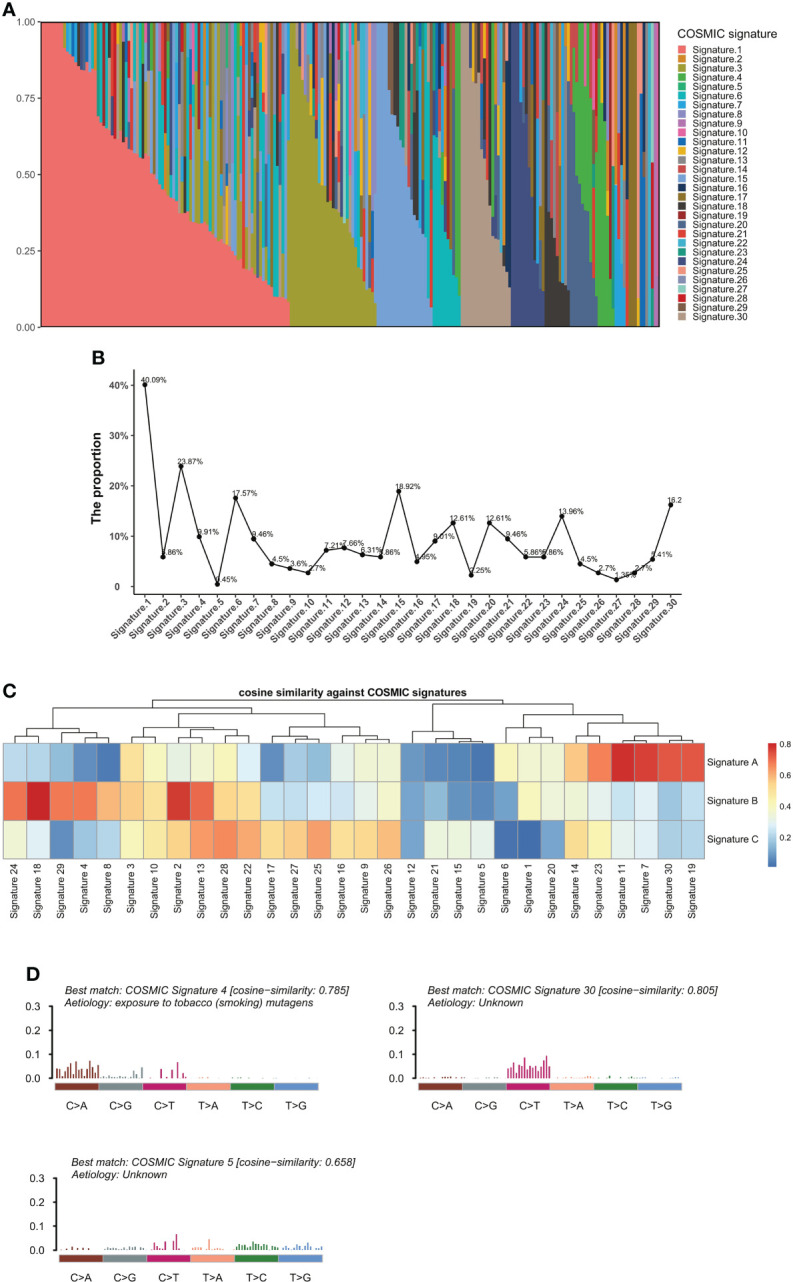
Analyses of mutational signatures in BTC patients. **(A)** Contribution of the 30 mutational signatures from the COSMIC among BTC patients. **(B)** The proportion of the 30 COSMIC mutational signatures in BTC patients. **(C)** Cosine similarities between three identified mutational signatures and COSMIC signatures. **(D)** Signature A, Signature B, and Signature C identified from BTC samples are linked to COSMIC Signature 4, Signature 30, and Signature 5, respectively. BTC, biliary tract cancer; COSMIC, Catalogue of Somatic Mutations in Cancer.

**Figure 9:**
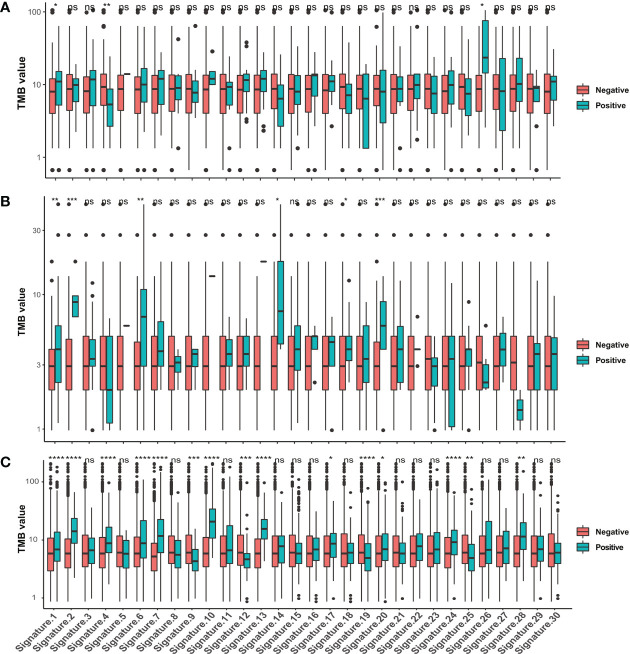
Correlations between TMB values and the 30 COSMIC mutational signatures. Comparison of TMB values in patients with and without COSMIC mutational signatures in **(A)** our BTC cohort, **(B)** the MSKCC BTC cohort, and **(C)** the MSKCC pan-cancer cohort. *p < 0.05; **p < 0.01; ***p < 0.001; ****p < 0.0001. TMB, tumor mutational burden; COSMIC, Catalogue of Somatic Mutations in Cancer; BTC, biliary tract cancer; MSKCC, Memorial Sloan Kettering Cancer Center. ns, no significance.

**Figure 10:**
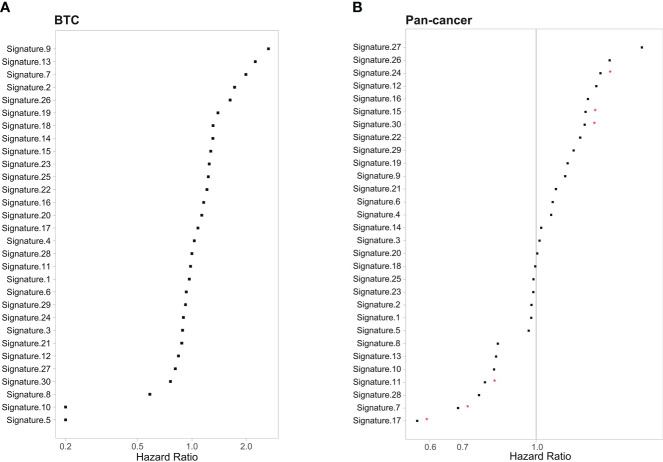
Correlation between the 30 COSMIC mutational signature and survival in **(A)** MSKCC BTC patients and **(B)** MSKCC pan-cancer patients. *p < 0.05. COSMIC, Catalogue of Somatic Mutations in Cancer; MSKCC, Memorial Sloan Kettering Cancer Center; BTC, biliary tract cancer.

To define the mutational signatures operative in BTC, we analyzed the proportion of 30 mutational signatures in patients from the Catalogue of Somatic Mutations in Cancer (COSMIC) database. In Chinese BTC patients, Signature 1 is the most prevalent one (**Figures 8A,B**, **Supplementary Table 4**). We extracted three mutational signatures (Signature A, Signature B, and Signature C) of our cohort by using non-negative matrix factorization (NMF) analysis, and they showed high similarity with the 30 human cancer mutational signatures in the COSMIC database (**Figure 8C**). Among them, Signature A showed a strong correlation with Signature 4, with a cosine correlation similarity of 0.785 (**Figure 8D**), suggesting that smoking may increase the risk of BTC, which has been confirmed in a previous study (30). Signature B and Signature C were correlated with Signature 30 (correlation similarity of 0.805) and Signature 5 (correlation similarity of 0.658), respectively (**Figure 8D**). These results demonstrate the reliability of mutational signature analysis using sequencing data from cancer-related gene panels. Subsequently, we analyzed the correlation between the COSMIC mutational signatures and the clinical characteristics of BTC. TMB value was significantly higher in patients with Signature 1, 4, and or 26 (**Figure 9A**). Signatures 1, 4, and 26 were associated with the age at diagnosis, smoking, and defective DNA mismatch repair (dMMR), respectively. Subsequently, we analyzed the mutational signatures of BTC and pan-cancer patients in the MSKCC database and found that the TMB level of patients with Signature 1 was significantly higher than those without (**Figure 9B**). Meanwhile, the TMB level was also significantly higher in patients with Signature 1 and/or Signature 4 in pan-cancer, similar to the results in our BTC cohort (**Figure 9C**). Although there was no significant difference in TMB level between patients with Signature 26 and without, an elevation trend in it was noticed (**Figure 9C**). These mutational signatures were not significantly associated with the survival of MSKCC patients (**Figure 10A**). Subsequently, we further evaluated the relationship between overall survival and mutational signature of ICI-treated pan-cancer patients in the MSKCC database, but found no significant difference in overall survival between patients with and without Signature 1, Signature 4, or Signature 26 (**Figure 10B**).

## Discussion

Surgery remains the cornerstone and curative treatment for BTC patients, but most BTC patients are unresectable at diagnosis and have intrahepatic sites, lymph nodes, or peritoneum metastasis (31). For advanced BTC patients, gemcitabine-based chemotherapies are the mostly applied systemic chemotherapy regimens, but the response rate and efficacy are generally poor (13, 32–35). Therefore, there are urgent needs to develop novel and more effective therapies for BTC, and recently, precision medicine and research have shown promising efficacy (36–38). The success of BTC precision medicine is linked to the better understanding of its molecular biology, but unfortunately, the genomic profile of Chinese BTC patients is still unclarified. To this end, we conducted a study that enrolled 382 Chinese BTC patients to comprehensively investigate their genomic feature to provide foundation for the clinical precision medicine and translational research in the future.

Further analysis of the different histological subtypes in our cohort revealed that while there were shared genomic features across ICC, ECC, and GBC, some key differences remain. For example, *APC*, *SMAD4*, and *TP53* were most prevalent in ICC, ECC, and GBC, respectively. Previous studies, in both Chinese and Western BTC patients, have also confirmed that *SMAD4* and *TP53* alterations were enriched in ECC and GBC, respectively (39, 40). APC, a known tumor suppressor gene and key regulator of the Wnt signaling pathway, remains unknown in BTC (41). Inactivation of the *APC* gene leads to disruption of β-catenin degradation, allowing free β-catenin to accumulate in the cytoplasm and translocate into the nucleus; as it was widely detected in the BTC cells, this was indicated as an early event for the carcinogenesis of BTC (42). Meanwhile, we found that *TP53* and *CREBBP* alterations were enriched in early-stage BTC patients, which contradicted the result from the previous study (43, 44). This difference may be contributed by relatively small sample sizes in their studies and difference in the patient characteristics. Additionally, we found that *NCOR1* alterations were more enriched in advanced BTC patients, which is in concordance with the result in the ICGC cohort (ratio of patients in advanced stages, mut vs. wt: 88.89% vs. 59.95%, *p* < 0.01) (45). However, little is known about the function of *NCOR1* alteration in BTC, which merited further exploration. Studies had shown that *NCOR1* can regulate hematopoiesis-related genes, and the decreased expression of hematopoietic-related gene promyelocytic leukemia zinc-finger (PLZF) in GBC was associated with advanced TNM stage, distant metastasis, and shorter overall survival (46, 47). We also noticed that BTC patients older than 60 years were more prone to have *DNMT3A* alteration, which is a hallmark of clonal hematopoiesis increasing with age (48). These findings may facilitate clinical trials or drug development targeted at these biomarkers in specific subtypes or characteristics of Chinese BTC patients.

Epidemiological risk factors for BTC include chronic inflammation (due to biliary and gallbladder stones, primary sclerosing cholangitis, liver flukes, and viral hepatitis), developmental abnormalities, and other environmental or metabolic factors such as diabetes, smoking, and drinking (49–51). Due to the prevalence of hepatitis virus (HBV/HCV) infection and liver fluke infection in China, the incidence of cholangiocarcinoma in China is high (52–54). Of note, in this study, the prevalence of *TP53*, *KRAS*, *IDH1*, *KMT2C*, and *SMAD4* was significantly different between the Chinese and Western BTC patients, which were also supported by other studies (38). Both *TP53* and *KRAS* alterations have been shown to be significantly associated with inferior survival outcomes in BTC (45, 55, 56). In addition, the prevalence of all actionable alterations in our BTC cohort is similar to that of the Western cohort with only one outlier, that *IDH1* was significantly more prevalent in the Western BTC patients. These differences in genetic alterations between different ethnic groups underscore the importance of genomic research and individual precision medicine. Except for the ethnic difference, the histology subtype is another factor determining the prevalence of actionable alterations. For example, in our cohort, *IDH1* alterations were mainly enriched in ICC patients (ICC vs. ECC vs. GBC, 2.8% vs. 1.5%% vs. 0.9%%), which was in concordance with a previous finding that *IDH1* alterations have been reported in approximately 13% of ICC patients but were rare in other histological subtypes of BTC (57). It is worth considering that the sample size and proportion of ICC in this study were relatively low, which may be a potential reason for the significantly lower prevalence of *IDH1* alterations in Chinese BTC patients than in Western BTC patients. Ivosidenib, an inhibitor of *IDH1*, effectively improved the median progression-free survival (2.7 months vs. 1.4 months, *p* < 0.0001) of patients (16). Another notable alteration is the *FGFR2* fusions or rearrangements, mainly occurring in about 20% of ICC patients but is rarely found in ECC (58). Pemigatinib is the first FDA-approved targeted drug for the treatment of previously treated patients with locally advanced or metastatic BTC harboring *FGFR2* fusions or rearrangements. The objective response rate of this drug in the second-line treatment of BTC patients was 35.5% (14, 59). About a year later, the FDA also approved infigratinib for the treatment of BTC with the same genomic alteration (60). Among patients treated with infigratinib, the objective response rate was 23%, the median progression-free survival was 7.3 months, and the median overall survival was 12.2 months. Unfortunately, our study did not include the detection of *FGFR2* structural variants, so we did not compare the difference in the prevalence of *FGFR2* fusions between Chinese and Western BTC patients, albeit we found that the *FGFR2* genomic alteration was more prevalent in ICC (ICC vs. ECC vs. GBC, 1.4% vs. 0.5% vs. 0). Activating *BRAF* alterations at the V600E locus is a well-known driver in pan-cancer and FDA-approved therapeutic targets for colorectal cancer, melanoma, non-small cell lung cancer, and anaplastic thyroid cancer. Though it was reported that *BRAF* alterations occurred in 5.0%–7.0% of Western BTC patients (61), the prevalence in our cohort was much lower (1.3%), consistent with the prevalence in another Chinese cohort (1.55%) (39). In a phase II multicenter basket trial, patients with advanced *BRAF*-V600E-mutated rare cancers, including biliary tract cancer, were treated with the *BRAF* inhibitor dabrafenib in combination with the *MEK* inhibitor trametinib (20), and encouraging antitumor effects, including 36% of cases achieving partial responses, a median progression-free survival of 9.2 months, and an overall survival of 11.7 months, were noticed (20). In addition, patients with several *BRAF*-V600-mutated cancer types were treated with another BRAF inhibitor (vemurafenib) and achieved similar response rates, with 2 out of 9 BTC patients (22.0%) having partial responses (62). It is widely known that *ERBB2* can be activated by overexpression, amplification, or alteration in multiple cancers, for instance, breast cancer, bladder cancer, and lung cancer, which has also been identified in patients with BTC. In ECC and GBC of our cohort, *ERBB2* alterations occurred in up to 2.6%–8.5% of cases, whereas its prevalence in ICC is much lower (1.4%), consistent with corresponding data reported in the Western cohort (63, 64). In a cohort of seven BTC patients, two patients had objective responses and three patients experienced prolonged stable disease after treating with trastuzumab plus pertuzumab (65). In another basket trial of patients with *ERBB2* or *ERBB3* alterations, two of nine BTC patients treated with neratinib achieved partial responses (66). However, more research is needed to determine the efficacy of *ERBB2*-targeted therapy as monotherapy or combination therapy in *ERBB2*-activated BTC patients. These genetic alterations showed a preference for histological subtypes, suggesting that ICC, ECC, and GBC may have different tumorigenesis and development mechanisms.

The heritable feature, particularly the cancer-predisposition genes associated with BTC, remains unknown. In this study, 10.5% of unselected Chinese BTC patients harbored P/LP germline alterations, which is approximate to recently published data from another Chinese cohort (12.0%) (39) and a Japanese BTC cohort (11.0%) by Wardell and his colleagues (40). In addition, an MSKCC cohort study showed that 16% (21/131) of BTC patients had pathogenic germline alterations, with the highest proportion of patients harboring *BRCA2* germline alterations (29), which were in concordance with our findings. Though previous studies suggested that Lynch syndrome, an autosomal dominant disease characterized by germline MMR alteration, is associated with an increasing lifetime risk of BTC (67), we found that the Lynch syndrome-related BTC was rare (0.53%) in Chinese patients. It is noteworthy that our study was the first time that identified *BLM*, *WRN*, *XPA*, and *RAD54L* germline P/LP alterations in BTC patients, supporting consideration of expanded genetic testing for those with positive family cancer history or early-onset disease (below 45 years old).

In recent years, the advent of ICIs has revolutionized the treatment prospects for some malignant tumors, and these drugs are also applied as monotherapy or in combination with other anticancer drugs to treat advanced BTC patients (68). However, the clinical benefit of immunotherapy appears to be limited to a small subset of BTC patients; hence, identifying reliable predictors of immunotherapy response is a major challenge. TMB-H has been reported as a predictive marker for immunotherapy or as a prognostic marker for various tumor types. Recently, Kim et al. analyzed the correlation between TMB status and ICI efficacy and showed that the efficacy and median progression-free survival of ICIs were significantly different between TMB-H and non-TMB-H patients (69). This finding suggests that TMB-H is a novel biomarker for predicting tumor response to ICIs in advanced BTC patients (69). In our study, we found that delDDR was associated with TMB value, suggesting that BTC patients with delDDR-mt were more likely to benefit from immunotherapy. In addition, Brandi et al. found differences in the proportion of TMB-H in different histological subtypes of BTC, with the highest proportion of TMB-H patients in GBC (68). This is consistent with our findings. Notably, in the dMMR or MSI-H subset of BTC, ICI treatment has shown success. In a seminal study of pembrolizumab across 12 tumor types with dMMR, four BTC cases were included. These patients achieved a 100% disease control rate, including one patient achieving complete response and three patients achieving stable disease (70). Our data showed that TMB levels are significantly higher in MSI-H patients; thus, BTC patients with TMB-H and MSI-H are more likely to benefit from immunotherapy, but more studies are needed for further validation. More importantly, we found that TMB levels were significantly higher in BTC patients with Signature 1, Signature 4, and Signature 26. Although the survival of patients with the aforementioned mutational signature in pan-cancer was not significantly improved after immunotherapy, it is worth further exploration in BTC to develop additional treatments.

In conclusion, our study comprehensively revealed the characteristics of the alterations in Chinese BTC, thereby improving our understanding of the mutational diversity of different histological subtypes of BTC. The findings would facilitate the identification of potential diagnostic and therapeutic biomarkers and provide the basis for genome-targeted strategies and clinical trials.

## Data availability statement

The original contributions presented in the study are included in the article/**Supplementary Material**. Further inquiries can be directed to the corresponding author.

## Ethics statement

This study was reviewed and approved by Medical Ethics Committee of Tianjin Medical University Cancer Institute & Hospital, National Clinical Research Center for Cancer. The patients/participants provided their written informed consent to participate in this study.

## Author contributions

HY: data collection and drafting of the manuscript, YX: drafting and revision, WG: data analysis, ML: data collection, JH: data collection, XD: sequencing data analysis, WX: design of this work and data analysis. All authors contributed to the article and approved the submitted version.

## Funding

This work was supported by the China Health Promotion Foundation (No: XM-2018-011-0006-01), Beijing Health Prevention and Therapy Association (No: IZ 2021-1001), China Health and Medical Development Foundation (No: GQ-KK-2021-001), and Tianjin Medical University Cancer Institute and Hospital (No: Pharmacology Spontaneous 2021-02).

## Acknowledgments

We thank all participating members of Tianjin Medical University Cancer Institute & Hospital in this research, as well as the technical assistance of Lifehealthcare Clinical Laboratory. This work was supported by Cancer Biobank of Tianjin Medical University Cancer Institute & Hospital.

## Conflict of interest

Author XD was employed by Lifehealthcare Clinical Laboratory.

The remaining authors declare that the research was conducted in the absence of any commercial or financial relationships that could be construed as a potential conflict of interest.

## Publisher’s note

All claims expressed in this article are solely those of the authors and do not necessarily represent those of their affiliated organizations, or those of the publisher, the editors and the reviewers. Any product that may be evaluated in this article, or claim that may be made by its manufacturer, is not guaranteed or endorsed by the publisher.

## Supplementary material

Supplementary Figure 1 Locations of TP53, KRAS, IDH1/2, NRAS, BRAF, KMT2C/D, AR, and NCOR1 alterations in the Chinese cohort. The different colors of the dots represent different alteration types.

Supplementary Figure 2 Differences in somatic alterations were compared between Chinese and MSKCC cohorts based on plasma samples. **(A)** ICC subtype. **(B)** ECC subtype. **(C)** GBC subtype. Differences in somatic alterations were compared between **(D)** Chinese and MSKCC cohorts, **(E)** Chinese and TCGA cohorts, and **(F)** Chinese and other Chinese (Nat Commun 2014) cohorts based on tumor samples. MSKCC, Memorial Sloan Kettering Cancer Center; ICC, intrahepatic cholangiocarcinoma; ECC, extrahepatic cholangiocarcinoma; GBC, gallbladder carcinoma; TCGA, The Cancer Genome Atlas.

## References

[1] Shaib Y El-Serag HB. The epidemiology of cholangiocarcinoma. Semin Liver Dis (2004) 24(2):115–25. doi: 10.1055/s-2004-828889 15192785

[2] Tella SH Kommalapati A Borad MJ Mahipal A. Second-line therapies in advanced biliary tract cancers. Lancet Oncol (2020) 21(1):e29–41. doi: 10.1016/S1470-2045(19)30733-8 31908303

[3] Lamarca A Barriuso J McNamara MG Valle JW. Molecular targeted therapies: Ready for "prime time" in biliary tract cancer. J Hepatol (2020) 73(1):170–85. doi: 10.1016/j.jhep.2020.03.007 32171892

[4] Patel T. Increasing incidence and mortality of primary intrahepatic cholangiocarcinoma in the united states. Hepatol (Baltimore Md) (2001) 33(6):1353–7. doi: 10.1053/jhep.2001.25087 11391522

[5] Patel N Benipal B. Incidence of cholangiocarcinoma in the USA from 2001 to 2015: A US cancer statistics analysis of 50 states. Cureus (2019) 11(1):e3962. doi: 10.7759/cureus.3962 30956914 PMC6436669

[6] Anderson C Kim R. Adjuvant therapy for resected extrahepatic cholangiocarcinoma: a review of the literature and future directions. Cancer Treat Rev (2009) 35(4):322–7. doi: 10.1016/j.ctrv.2008.11.009 19147294

[7] DeOliveira ML Cunningham SC Cameron JL Kamangar F Winter JM Lillemoe KD. Cholangiocarcinoma: thirty-one-year experience with 564 patients at a single institution. Ann Surg (2007) 245(5):755–62. doi: 10.1097/01.sla.0000251366.62632.d3 PMC1877058 17457168

[8] Zheng Y Tu X Zhao P Jiang W Liu L Tong Z. A randomised phase II study of second-line XELIRI regimen versus irinotecan monotherapy in advanced biliary tract cancer patients progressed on gemcitabine and cisplatin. Br J Cancer (2018) 119(3):291–5. doi: 10.1038/s41416-018-0138-2 PMC6068158 29955136

[9] Lamarca A Hubner RA David Ryder W Valle JW. Second-line chemotherapy in advanced biliary cancer: a systematic review. Ann Oncol Off J Eur Soc Med Oncol (2014) 25(12):2328–38. doi: 10.1093/annonc/mdu162 24769639

[10] Brandi G Rizzo A Dall'Olio FG Felicani C Ercolani G Cescon M. Percutaneous radiofrequency ablation in intrahepatic cholangiocarcinoma: a retrospective single-center experience. Int J Hyperthermia Off J Eur Soc Hyperthermic Oncol North Am Hyperthermia Group (2020) 37(1):479–85. doi: 10.1080/02656736.2020.1763484 32396398

[11] Bridgewater JA Goodman KA Kalyan A Mulcahy MF. Biliary tract cancer: Epidemiology, radiotherapy, and molecular profiling. Am Soc Clin Oncol Educ Book Am Soc Clin Oncol Annu Meeting (2016) 35:e194–203. doi: 10.1200/EDBK_160831 27249723

[12] Jung SJ Woo SM Park HK Lee WJ Han MA Han SS. Patterns of initial disease recurrence after resection of biliary tract cancer. Oncology (2012) 83(2):83–90. doi: 10.1159/000339695 22777276

[13] Valle J Wasan H Palmer DH Cunningham D Anthoney A Maraveyas A. Cisplatin plus gemcitabine versus gemcitabine for biliary tract cancer. New Engl J Med (2010) 362(14):1273–81. doi: 10.1056/NEJMoa0908721 20375404

[14] Abou-Alfa GK Sahai V Hollebecque A Vaccaro G Melisi D Al-Rajabi R. Pemigatinib for previously treated, locally advanced or metastatic cholangiocarcinoma: a multicentre, open-label, phase 2 study. Lancet Oncol (2020) 21(5):671–84. doi: 10.1016/S1470-2045(20)30109-1 PMC8461541 32203698

[15] Makawita S KA-A G Roychowdhury S Sadeghi S Borbath I Goyal L. Infigratinib in patients with advanced cholangiocarcinoma with FGFR2 gene fusions/translocations: the PROOF 301 trial. Future Oncol (Lond Engl) (2020) 16(30):2375–84. doi: 10.2217/fon-2020-0299 32580579

[16] Abou-Alfa GK Macarulla T Javle MM Kelley RK Lubner SJ Adeva J. Ivosidenib in IDH1-mutant, chemotherapy-refractory cholangiocarcinoma (ClarIDHy): a multicentre, randomised, double-blind, placebo-controlled, phase 3 study. Lancet Oncol (2020) 21(6):796–807. doi: 10.1016/S1470-2045(20)30157-1 32416072 PMC7523268

[17] Drilon A Laetsch TW Kummar S DuBois SG Lassen UN Demetri GD. Efficacy of larotrectinib in TRK fusion-positive cancers in adults and children. New Engl J Med (2018) 378(8):731–9. doi: 10.1056/NEJMoa1714448 PMC5857389 29466156

[18] Doebele RC Drilon A Paz-Ares L Siena S Shaw AT Farago AF. Entrectinib in patients with advanced or metastatic NTRK fusion-positive solid tumours: integrated analysis of three phase 1-2 trials. Lancet Oncol (2020) 21(2):271–82. doi: 10.1016/S1470-2045(19)30691-6 PMC7461630 31838007

[19] Salama AKS Li S Macrae ER Park JI Mitchell EP Zwiebel JA. Dabrafenib and trametinib in patients with tumors with BRAF(V600E) mutations: Results of the NCI-MATCH trial subprotocol h. J Clin Oncol Off J Am Soc Clin Oncol (2020) 38(33):3895–904. doi: 10.1200/JCO.20.00762 PMC7676884 32758030

[20] Subbiah V Lassen U Élez E Italiano A Curigliano G Javle M. Dabrafenib plus trametinib in patients with BRAF(V600E)-mutated biliary tract cancer (ROAR): a phase 2, open-label, single-arm, multicentre basket trial. Lancet Oncol (2020) 21(9):1234–43. doi: 10.1016/S1470-2045(20)30321-1 32818466

[21] Cao J Chen L Li H Chen H Yao J Mu S. An accurate and comprehensive clinical sequencing assay for cancer targeted and immunotherapies. oncologist (2019) 24(12):e1294–302. doi: 10.1634/theoncologist.2019-0236 PMC6975945 31409745

[22] Salipante SJ Scroggins SM Hampel HL Turner EH Pritchard CC. Microsatellite instability detection by next generation sequencing. Clin Chem (2014) 60(9):1192–9. doi: 10.1373/clinchem.2014.223677 24987110

[23] Chakravarty D Gao J Phillips SM Kundra R Zhang H Wang J. OncoKB: A precision oncology knowledge base. JCO Precis Oncol (2017) 2017:PO.17.00011. doi: 10.1200/PO.17.00011 PMC5586540 28890946

[24] Lin A Qiu Z Zhang J Luo P. Effect of NCOR1 mutations on immune microenvironment and efficacy of immune checkpoint inhibitors in patient with bladder cancer. Front Immunol (2021) 12:630773. doi: 10.3389/fimmu.2021.630773 33763074 PMC7982737

[25] St-Jean S De Castro AC Lecours M Jones C Rivard N Rodier F. NCOR1 sustains colorectal cancer cell growth and protects against cellular senescence. Cancers (2021) 13(17):4414. doi: 10.3390/cancers13174414 34503224 PMC8430780

[26] Lian J Xu C Chen X Huang S Wu D. Histone methyltransferase KMT2C plays an oncogenic role in prostate cancer. J Cancer Res Clin Oncol (2022) 148(7):1627–40. doi: 10.1007/s00432-022-03968-5 PMC11800848 35322299

[27] Limberger T Schlederer M Trachtová K Garces de Los Fayos Alonso I Yang J Högler S. KMT2C methyltransferase domain regulated INK4A expression suppresses prostate cancer metastasis. Mol Cancer (2022) 21(1):89. doi: 10.1186/s12943-022-01542-8 35354467 PMC8966196

[28] Zhang J Dominguez-Sola D Hussein S Lee JE Holmes AB Bansal M. Disruption of KMT2D perturbs germinal center b cell development and promotes lymphomagenesis. Nat Med (2015) 21(10):1190–8. doi: 10.1038/nm.3940 PMC5145002 26366712

[29] Maynard H Stadler ZK Berger MF Solit DB Ly M Lowery MA. Germline alterations in patients with biliary tract cancers: A spectrum of significant and previously underappreciated findings. Cancer (2020) 126(9):1995–2002. doi: 10.1002/cncr.32740 32012241 PMC7584349

[30] Hou L Jiang J Liu B Han W Wu Y Zou X. Is exposure to tobacco associated with extrahepatic cholangiocarcinoma epidemics? a retrospective proportional mortality study in China. BMC Cancer (2019) 19(1):348. doi: 10.1186/s12885-019-5484-9 30975121 PMC6458766

[31] Valle JW. Advances in the treatment of metastatic or unresectable biliary tract cancer. Ann Oncol Off J Eur Soc Med Oncol (2010) 21 (Suppl 7):vii345–348. doi: 10.1093/annonc/mdq420 20943640

[32] Okusaka T Nakachi K Fukutomi A Mizuno N Ohkawa S Funakoshi A. Gemcitabine alone or in combination with cisplatin in patients with biliary tract cancer: a comparative multicentre study in Japan. Br J Cancer (2010) 103(4):469–74. doi: 10.1038/sj.bjc.6605779 PMC2939781 20628385

[33] André T Tournigand C Rosmorduc O Provent S Maindrault-Goebel F Avenin D. Gemcitabine combined with oxaliplatin (GEMOX) in advanced biliary tract adenocarcinoma: a GERCOR study. Ann Oncol Off J Eur Soc Med Oncol (2004) 15(9):1339–43. doi: 10.1093/annonc/mdh351 15319238

[34] Lee J Park SH Chang HM Kim JS Choi HJ Lee MA. Gemcitabine and oxaliplatin with or without erlotinib in advanced biliary-tract cancer: a multicentre, open-label, randomised, phase 3 study. Lancet Oncol (2012) 13(2):181–8. doi: 10.1016/S1470-2045(11)70301-1 22192731

[35] Shroff RT Javle MM Xiao L Kaseb AO Varadhachary GR Wolff RA. Gemcitabine, cisplatin, and nab-paclitaxel for the treatment of advanced biliary tract cancers: A phase 2 clinical trial. JAMA Oncol (2019) 5(6):824–30. doi: 10.1001/jamaoncol.2019.0270 PMC6567834 30998813

[36] Sicklick JK Kato S Okamura R Schwaederle M Hahn ME Williams CB. Molecular profiling of cancer patients enables personalized combination therapy: the I-PREDICT study. Nat Med (2019) 25(5):744–50. doi: 10.1038/s41591-019-0407-5 PMC6553618 31011206

[37] Rodon J Soria JC Berger R Miller WH Rubin E Kugel A. Genomic and transcriptomic profiling expands precision cancer medicine: the WINTHER trial. Nat Med (2019) 25(5):751–8. doi: 10.1038/s41591-019-0424-4 PMC6599610 31011205

[38] Schwaederle M Zhao M Lee JJ Eggermont AM Schilsky RL Mendelsohn J. Impact of precision medicine in diverse cancers: A meta-analysis of phase II clinical trials. J Clin Oncol Off J Am Soc Clin Oncol (2015) 33(32):3817–25. doi: 10.1200/JCO.2015.61.5997 PMC4737863 26304871

[39] Lin J Cao Y Yang X Li G Shi Y Wang D. Mutational spectrum and precision oncology for biliary tract carcinoma. Theranostics (2021) 11(10):4585–98. doi: 10.7150/thno.56539 PMC7978308 33754015

[40] Wardell CP Fujita M Yamada T Simbolo M Fassan M Karlic R. Genomic characterization of biliary tract cancers identifies driver genes and predisposing mutations. J Hepatol (2018) 68(5):959–69. doi: 10.1016/j.jhep.2018.01.009 29360550

[41] Fodde R. The APC gene in colorectal cancer. Eur J Cancer (Oxford Engl 1990) (2002) 38(7):867–71. doi: 10.1016/S0959-8049(02)00040-0 11978510

[42] Maroni L Pierantonelli I Banales JM Benedetti A Marzioni M. The significance of genetics for cholangiocarcinoma development. Ann Trans Med (2012) 1(3):7. doi: 10.3978/j.issn.2305-5839.2012.10.04 PMC4200671 25332972

[43] Tian W Hu W Shi X Liu P Ma X Zhao W. Comprehensive genomic profile of cholangiocarcinomas in China. Oncol Lett (2020) 19(4):3101–10. doi: 10.3892/ol.2020.11429 PMC7074170 32256810

[44] Chen X Wang D Liu J Qiu J Zhou J Ying J. Genomic alterations in biliary tract cancer predict prognosis and immunotherapy outcomes. J Immunother Cancer (2021) 9(11):e003214. doi: 10.1136/jitc-2021-003214 34795005 PMC8603283

[45] Jusakul A Cutcutache I Yong CH Lim JQ Huang MN Padmanabhan N. Whole-genome and epigenomic landscapes of etiologically distinct subtypes of cholangiocarcinoma. Cancer Discov (2017) 7(10):1116–35. doi: 10.1158/2159-8290.CD-17-0368 PMC5628134 28667006

[46] Wan X Liu L Zhou P Hui X He Q Yu F. The nuclear receptor corepressor NCoR1 regulates hematopoiesis and leukemogenesis. vivo Blood Adv (2019) 3(4):644–57. doi: 10.1182/bloodadvances.2018022756 PMC6391666 30804018

[47] Shen H Zhan M Zhang Y Huang S Xu S Huang X. PLZF inhibits proliferation and metastasis of gallbladder cancer by regulating IFIT2. Cell Death Dis (2018) 9(2):71. doi: 10.1038/s41419-017-0107-3 29358655 PMC5833736

[48] Marina S Anne Kathrin L Stefanie G Christian R Stephen K Sina S. Hotspot DNMT3A mutations in clonal hematopoiesis and acute myeloid leukemia sensitize cells to azacytidine. via Viral Mimicry Response Nat Cancer (2021) 2(5):527–44. doi: 10.1038/s43018-021-00213-9 35122024

[49] Tyson GL El-Serag HB. Risk factors for cholangiocarcinoma. Hepatol (Baltimore Md) (2011) 54(1):173–84. doi: 10.1002/hep.24351 PMC3125451 21488076

[50] Rizvi S Gores GJ. Pathogenesis, diagnosis, and management of cholangiocarcinoma. Gastroenterology (2013) 145(6):1215–29. doi: 10.1053/j.gastro.2013.10.013 PMC3862291 24140396

[51] Clements O Eliahoo J Kim JU Taylor-Robinson SD Khan SA. Risk factors for intrahepatic and extrahepatic cholangiocarcinoma: A systematic review and meta-analysis. J Hepatol (2020) 72(1):95–103. doi: 10.1016/j.jhep.2019.09.007 31536748

[52] Athauda A Fong C Lau DK Javle M Abou-Alfa GK Morizane C. Broadening the therapeutic horizon of advanced biliary tract cancer through molecular characterisation. Cancer Treat Rev (2020) 86:101998. doi: 10.1016/j.ctrv.2020.101998 32203843 PMC8222858

[53] Valle JW Kelley RK Nervi B Oh DY Zhu AX. Biliary tract cancer. Lancet (Lond Engl) (2021) 397(10272):428–44. doi: 10.1016/S0140-6736(21)00153-7 33516341

[54] Zhang H Zhu B Zhang H Liang J Zeng W. HBV infection status and the risk of cholangiocarcinoma in Asia: A meta-analysis. BioMed Res Int (2016) 2016:3417976. doi: 10.1155/2016/3417976 27999794 PMC5141322

[55] Nakamura H Arai Y Totoki Y Shirota T Elzawahry A Kato M. Genomic spectra of biliary tract cancer. Nat Genet (2015) 47(9):1003–10. doi: 10.1038/ng.3375 26258846

[56] Chae H Kim D Yoo C Kim KP Jeong JH Chang HM. Therapeutic relevance of targeted sequencing in management of patients with advanced biliary tract cancer: DNA damage repair gene mutations as a predictive biomarker. Eur J Cancer (Oxford Engl 1990) (2019) 120:31–9. doi: 10.1016/j.ejca.2019.07.022 31476489

[57] Boscoe AN Rolland C Kelley RK. Frequency and prognostic significance of isocitrate dehydrogenase 1 mutations in cholangiocarcinoma: a systematic literature review. J Gastrointestinal Oncol (2019) 10(4):751–65. doi: 10.21037/jgo.2019.03.10 PMC6657309 31392056

[58] Goyal L Shi L Liu LY Fece de la Cruz F Lennerz JK Raghavan S. TAS-120 overcomes resistance to ATP-competitive FGFR inhibitors in patients with FGFR2 fusion-positive intrahepatic cholangiocarcinoma. Cancer Discovery (2019) 9(8):1064–79. doi: 10.1158/2159-8290.CD-19-0182 PMC6677584 31109923

[59] Hoy SM. Pemigatinib: First approval. Drugs (2020) 80(9):923–9. doi: 10.1007/s40265-020-01330-y 32472305

[60] Javle M Roychowdhury S Kelley RK Sadeghi S Macarulla T Weiss KH. Infigratinib (BGJ398) in previously treated patients with advanced or metastatic cholangiocarcinoma with FGFR2 fusions or rearrangements: mature results from a multicentre, open-label, single-arm, phase 2 study. Lancet Gastroenterol Hepatol (2021) 6(10):803–15. doi: 10.1016/S2468-1253(21)00196-5 34358484

[61] Robertson S Hyder O Dodson R Nayar SK Poling J Beierl K. The frequency of KRAS and BRAF mutations in intrahepatic cholangiocarcinomas and their correlation with clinical outcome. Hum Pathol (2013) 44(12):2768–73. doi: 10.1016/j.humpath.2013.07.026 PMC3838441 24139215

[62] Subbiah V Puzanov I Blay JY Chau I Lockhart AC Raje NS. Pan-cancer efficacy of vemurafenib in BRAF (V600)-mutant non-melanoma cancers. Cancer Discovery (2020) 10(5):657–63. doi: 10.1158/2159-8290.CD-19-1265 PMC7196502 32029534

[63] Javle M Bekaii-Saab T Jain A Wang Y Kelley RK Wang K. Biliary cancer: Utility of next-generation sequencing for clinical management. Cancer (2016) 122(24):3838–47. doi: 10.1002/cncr.30254 27622582

[64] Valle JW Lamarca A Goyal L Barriuso J Zhu AX. New horizons for precision medicine in biliary tract cancers. Cancer Discov (2017) 7(9):943–62. doi: 10.1158/2159-8290.CD-17-0245 PMC5586506 28818953

[65] Hainsworth JD Meric-Bernstam F Swanton C Hurwitz H Spigel DR Sweeney C. Targeted therapy for advanced solid tumors on the basis of molecular profiles: Results from MyPathway, an open-label, phase IIa multiple basket study. J Clin Oncol Off J Am Soc Clin Oncol (2018) 36(6):536–42. doi: 10.1200/JCO.2017.75.3780 29320312

[66] Hyman DM Piha-Paul SA Won H Rodon J Saura C Shapiro GI. HER kinase inhibition in patients with HER2- and HER3-mutant cancers. Nature (2018) 554(7691):189–94. doi: 10.1038/nature25475 PMC5808581 29420467

[67] Cloyd J Chun Y Ikoma N Vauthey J Aloia T Cuddy A. Clinical and genetic implications of DNA mismatch repair deficiency in biliary tract cancers associated with lynch syndrome. J Gastrointestinal Cancer (2018) 49(1):93–6. doi: 10.1007/s12029-017-0040-9 PMC7703856 29238914

[68] Rizzo A Ricci AD Brandi G. PD-L1, TMB, MSI, and other predictors of response to immune checkpoint inhibitors in biliary tract cancer. Cancers (2021) 13(3):558. doi: 10.3390/cancers13030558 33535621 PMC7867133

[69] Kim H Kim H Kim R Jo H Kim HR Hong J. Tumor mutational burden as a biomarker for advanced biliary tract cancer. Technol Cancer Res Treat (2021) 20:15330338211062324. doi: 10.1177/15330338211062324 34855561 PMC8646759

[70] Le DT Durham JN Smith KN Wang H Bartlett BR Aulakh LK. Mismatch repair deficiency predicts response of solid tumors to PD-1 blockade. Sci (New York NY) (2017) 357(6349):409–13. doi: 10.1126/science.aan6733 PMC5576142 28596308

