# Supplementary figures and images for: Comprehensive germline and somatic genomic profiles of Chinese patients with biliary tract cancer
Source: Front Oncol. 2022 Aug 22;12:930611. doi: 10.3389/fonc.2022.930611 (PMC9441936; doi:10.3389/fonc.2022.930611)

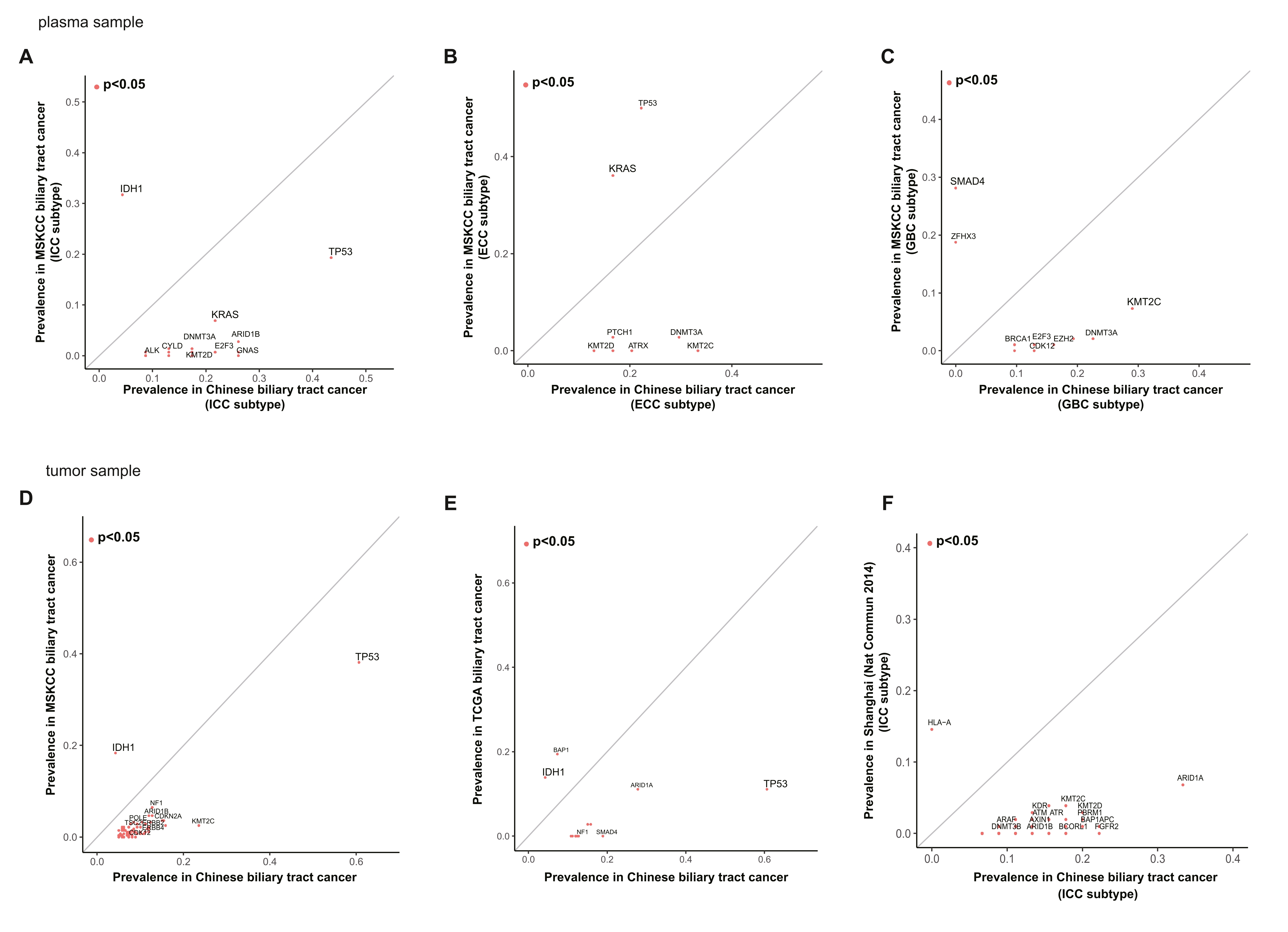

Supplement: Supplementary Figure 2 — Differences in somatic alterations were compared between Chinese and MSKCC cohorts based on plasma samples. (A) ICC subtype. (B) ECC subtype. (C) GBC subtype. Differences in somatic alterations were compared between (D) Chinese and MSKCC cohorts, (E) Chinese and TCGA cohorts, and (F) Chinese and other Chinese (Nat Commun 2014) cohorts based on tumor samples. MSKCC, Memorial Sloan Kettering Cancer Center; ICC, intrahepatic cholangiocarcinoma; ECC, extrahepatic cholangiocarcinoma; GBC, gallbladder carcinoma; TCGA, The Cancer Genome Atlas. [file Image_2.jpeg]
