# Supplementary material for: Comprehensive germline and somatic genomic profiles of Chinese patients with biliary tract cancer
Source: Front Oncol. 2022 Aug 22;12:930611. doi: 10.3389/fonc.2022.930611 (PMC9441936; doi:10.3389/fonc.2022.930611)
Supplement: Supplementary file 3 [file Table_1.docx]

**Supplemental Table 1**. The gene list of the 499-gene panel used for gene mutation detection in this study.

| *ABL1* | *CBFB* | *CASP8* | *CCND1* | *CSF1R* | *DNMT3A** | *FBXW7** | *GNAQ* | *IGF2* | *KMT2A* |
| --- | --- | --- | --- | --- | --- | --- | --- | --- | --- |
| *ABL2* | *CBL* | *CRLF2* | *CCND2* | *CSF3R* | *DNMT3B* | *FGF10* | *GNAS* | *IKBKE* | *KMT2B* |
| *ACVR1* | *AXL* | *CSDE1* | *CCND3* | *CTCF* | *DOT1L* | *FGF14* | *GOPC* | *IKZF1* | *KMT2C* |
| *ACVR1B* | *B2M** | *ESR1^&^* | *CCNE1* | *CTLA4* | *DROSHA* | *FGF19* | *GPR124* | *IL10* | *KMT2D* |
| *AGO2* | *BAP1** | *ETV1^&^* | *CD274* | *CTNNA1* | *DUSP4* | *FGF23* | *GREM1* | *IL7R* | *KNSTRN* |
| *AKT1* | *BARD1** | *ETV6^&^* | *CD276* | *CTNNB1** | *E2F3* | *FGF3* | *GRIN2A* | *INHBA* | *KRAS** |
| *AKT2* | *BCL10* | *EZH2** | *CD74^&^* | *CUL3* | *EED* | *FGF4* | *GRM3* | *INPP4A* | *LATS1* |
| *AKT3* | *BCL2* | *FAM175A* | *CD79A* | *CXCR4* | *EGF* | *FGF6* | *GSK3B* | *INPP4B* | *LATS2* |
| *ALK^&^* | *BCL2L1* | *FAM46C* | *CD79B* | *CYLD* | *EGFR** | *FGFR1** | *GSTA1* | *INPPL1* | *LMO1* |
| *ALOX12B* | *BCL2L11* | *FAM58A* | *CDC42* | *CYSLTR2* | *EIF1AX* | *FGFR2*^&^* | *H3F3A* | *INSR* | *LRP1B* |
| *AMER1* | *BCL2L2* | *FANCA** | *CDC73** | *DAXX* | *EIF4A2* | *FGFR3***^&^* | *H3F3B* | *IRF2* | *LRRK2* |
| *ANKRD11* | *BCL6* | *FANCC** | *CDH1** | *DDR2** | *ELF3* | *FGFR4** | *HDAC1* | *IRF4* | *LYN* |
| *APC** | *BCOR* | *FANCD2** | *CDK12** | *DICER1* | *EML4^&^* | *FH** | *HDAC4* | *IRS1* | *LZTR1* |
| *AR** | *BCORL1* | *FANCE** | *CDK4* | *DIS3* | *EP300* | *FLCN** | *HGF* | *IRS2* | *MAGI2* |
| *ARAF* | *BIRC3* | *FANCF** | *CDK6* | *DNAJB1* | *EPAS1* | *FLT1* | *HIST1H1C* | *JAK1** | *MALT1* |
| *ARFRP1* | *BLM** | *FANCG* | *CDK8* | *DNMT1* | *EPCAM** | *FLT3* | *HIST1H2BD* | *JAK2** | *MAP2K1** |
| *ARID1A** | *BMPR1A** | *FANCL** | *CDKN1A* | *ERG* | *EPHA3* | *FLT4* | *HIST1H3B* | *JAK3** | *MAP2K2** |
| *ARID1B* | *BRAF** | *FAS* | *CDKN1B* | *ERRFI1* | *EPHA5* | *FOLR3* | *HIST1H3G* | *JUN* | *MAP2K4* |
| *ARID2* | *BRCA1** | *FAT1* | *CDKN2A** | *GATA4* | *EPHA7* | *FOXA1** | *HLA-A* | *KAT6A* | *MAP3K1* |
| *ARID5B* | *BRCA2** | *PRKDC* | *CDKN2B** | *GATA6* | *EPHB1* | *FOXL2* | *HLA-B* | *KDM5A* | *MAP3K13* |
| *ASXL1* | *BRD3* | *PRSS8* | *CDKN2C* | *GID4* | *ERBB2* | *FOXO1* | *HNF1A* | *KDM5C** | *MAP3K14* |
| *ASXL2* | *BRD4* | *PTCH1** | *CEBPA* | *GLI1* | *ERBB3* | *FOXP1* | *HOXB13** | *KDM6A** | *MAPK1* |
| *ATM** | *BRIP1** | *PTCH2** | *CHD2* | *GNA11* | *ERBB4* | *FRS2* | *HRAS** | *KDR* | *MAPK3* |
| *ATR** | *BTG1* | *PTEN** | *CHD4* | *GNA13* | *ERCC1** | *FUBP1* | *HSD3B1** | *KEAP1* | *MAX** |
| *ATRX** | *BTK* | *PTPN11* | *CHEK1** | *IGF1* | *ERCC2** | *FYN* | *HSP90AA1* | *KEL* | *MCL1* |
| *AURKA* | *C11orf30* | *PTPRD* | *CHEK2** | *IGF1R* | *ERCC3** | *GABRA6* | *ID3* | *KIT* | *MDC1** |
| *AURKB* | *CALR* | *PTPRS* | *CIC* | *RAD51** | *ERCC4** | *GATA1* | *IDH1** | *KLF4* | *MDM2** |
| *AXIN1** | *CARD11* | *PTPRT* | *CREBBP** | *RAD51B** | *ERCC5** | *GATA2* | *IDH2** | *KLHL6* | *MDM4** |
| *AXIN2* | *CARM1* | *QKI* | *CRKL* | *RAD51C** | *ERF* | *GATA3* | *IFNGR1* | *TNFRSF14* | *MED12* |
| *WHSC1* | *RAD21* | *RAB35* | *RAF1** | *RAD51D** | *TP63* | *TSHR* | *TSC1** | *TOP1* | *VEGFA* |
| *WHSC1L1* | *RAD50** | *RAC1* | *RANBP2* | *RAD52** | *TRAF2* | *TYMS* | *TSC2** | *TOP2A* | *VHL** |
| *SNCAIP* | *SOCS1* | *SOS1* | *SOX10* | *SOX17* | *SOX2* | *SOX9* | *SPEN* | *SPOP* | *SPTA1* |
| *STAT5B* | *STK11** | *SUFU* | *SUZ12* | *SYK* | *TAF1* | *TAP1* | *TAP2* | *TBX3* | *TCEB1* |
| *STAT5A* | *SLX4* | *RARA* | *RIT1* | *RUNX1T1* | *YAP1* | *SHOC2* | *TP53BP1** | *ZNF703* | *PGR* |
| *TET2* | *SMAD2* | *RASA1* | *RNF43* | *TGFBR1* | *YES1* | *SHQ1* | *PRDM14* | *PAK3* | *PHOX2B* |
| *RXRA* | *SMAD3* | *RB1** | *ROCK1* | *TGFBR2* | *ZBTB2* | *SLC19A1* | *PDGFRB* | *PAK7* | *PIK3C2B* |
| *RYBP* | *SMAD4** | *RBM10* | *ROS1* | *TMEM127* | *ZFHX3* | *SLIT2* | *TRAF7* | *PALB2** | *PIK3C2G* |
| *SDHA** | *SMARCA4* | *RECQL* | *RPS6KA4* | *TMPRSS2* | *ZNF217* | *STAT3* | *MEF2B* | *PARK2* | *PIK3C3* |
| *SDHAF2* | *SMARCB1** | *RECQL4** | *RPS6KB1* | *TNFAIP3* | *STAT4* | *TERT^&^* | *MEN1** | *PARP1** | *PIK3CA** |
| *SDHB** | *SMARCD1* | *REL* | *RPTOR* | *TP53** | *TET1* | *MSH2** | *MET* | *PARP2* | *PIK3CB* |
| *SDHC** | *SMO* | *RET^&^* | *RRAGC* | *MYOD1* | *MUTYH** | *MSH3** | *MGA* | *PAX5* | *PIK3CD* |
| *SDHD** | *WWTR1* | *RFWD2* | *RRAS2* | *NAT2* | *MYC* | *MSH6** | *MITF** | *PBRM1** | *PIK3CG* |
| *SETD2* | *XIAP* | *RHEB* | *RRM1* | *NBN** | *MYCL* | *MSI2* | *MLH1** | *PDCD1* | *PIK3R1* |
| *SF3B1* | *XPO1* | *RHOA* | *RTEL1* | *NCOA3* | *MYCN* | *MST1R* | *MPL* | *PDCD1LG2* | *PIK3R2* |
| *SH2B3* | *XRCC2* | *RICTOR* | *RUNX1* | *NCOR1* | *MYD88* | *MTOR* | *MRE11A** | *PDGFRA* | *PIK3R3* |
| *NEGR1* | *NFKBIA* | *SRC* | *PMS2** | *PPM1D* | *SRSF2* | *RAD54L** | *PDK1* | *STAG2* | *NOTCH4* |
| *NF1** | *WISP3* | *TCF3* | *PNRC1* | *PPP2R1A** | *TCF7L2* | *U2AF1* | *PDPK1* | *TEK* | *NPM1* |
| *NF2** | *WT1* | *PIM1* | *POLD1** | *PPP6C* | *PREX2* | *NUP93* | *RAC2* | *NKX2-1* | *NRAS** |
| *NFE2L2* | *PMS1** | *PLCG2* | *POLE** | *PRDM1* | *PRKAR1A* | *OPRM1* | *NTRK3^&^* | *NOTCH1** | *NSD1* |
| *NTRK1^&^* | *NTRK2^&^* | *NOTCH3* | *PPARG* | *PRKD1* | *PRKCI* | *PAK1* | *NUF2* | *NOTCH2* |  |

* Genes with full exonic regions

& Genes with selected intronic regions

Unlabel genes are those with selected exonic regions
